# A Simulation Study of Acoustic-Assisted Tracking of Whales for Mark-Recapture Surveys

**DOI:** 10.1371/journal.pone.0095602

**Published:** 2014-05-14

**Authors:** David Peel, Brian S. Miller, Natalie Kelly, Steve Dawson, Elisabeth Slooten, Michael C. Double

**Affiliations:** 1 CSIRO Computational Informatics/Wealth from Oceans National Research Flagship, Castray Esplanade, Hobart, Tasmania, Australia; 2 Australian Marine Mammal Centre, Australian Antarctic Division, Department of the Environment, Channel Highway, Kingston, Australia; 3 University of Otago, Dunedin, New Zealand; University of Pavia, Italy

## Abstract

Collecting enough data to obtain reasonable abundance estimates of whales is often difficult, particularly when studying rare species. Passive acoustics can be used to detect whale sounds and are increasingly used to estimate whale abundance. Much of the existing effort centres on the use of acoustics to estimate abundance directly, e.g. analysing detections in a distance sampling framework. Here, we focus on acoustics as a tool incorporated within mark-recapture surveys. In this context, acoustic tools are used to detect and track whales, which are then photographed or biopsied to provide data for mark-recapture analyses. The purpose of incorporating acoustics is to increase the encounter rate beyond using visual searching only. While this general approach is not new, its utility is rarely quantified. This paper predicts the “acoustically-assisted” encounter rate using a discrete-time individual-based simulation of whales and survey vessel. We validate the simulation framework using existing data from studies of sperm whales. We then use the framework to predict potential encounter rates in a study of Antarctic blue whales. We also investigate the effects of a number of the key parameters on encounter rate. Mean encounter rates from the simulation of sperm whales matched well with empirical data. Variance of encounter rate, however, was underestimated. The simulation of Antarctic blue whales found that passive acoustics should provide a 1.7–3.0 fold increase in encounter rate over visual-only methods. Encounter rate was most sensitive to acoustic detection range, followed by vocalisation rate. During survey planning and design, some indication of the relationship between expected sample size and effort is paramount; this simulation framework can be used to predict encounter rates and establish this relationship. For a case in point, the simulation framework indicates unequivocally that real-time acoustic tracking should be considered for quantifying the abundance of Antarctic blue whales via mark-recapture methods.

## Introduction

Knowledge of population abundance is critical to the management and conservation of whales. The most common tools used to measure abundance are line-transect distance sampling (see [1], [2]) and mark-recapture surveys (see [3], and for example, [4], [5]). However, when species are rare, and/or survey effort is limited, it can be difficult to encounter enough animals to estimate abundance with adequate precision. For many whale species, passive acoustics has a detection range an order of magnitude greater than that of visual observations (e.g. [6]). Therefore, using passive acoustics (henceforth referred to only as acoustics) to find whales may substantially increase encounter rate (i.e. sample size and hence increased precision).

In the context of abundance assessment, most studies using acoustic tools have focused on estimating abundance directly in a line-transect (e.g. [6]) or point transect (e.g. [7]) framework. In these approaches acoustic detections are often used in an analogous way to sightings made by visual observers. This paper examines an alternate use of acoustics in abundance estimation, namely as a tool to detect and assist in tracking down animals in a mark-recapture survey [8]–[10].

Acoustically-assisted mark-recapture is not a new idea, and is often described as being complementary to visual survey methods; efficiency and low cost often listed as benefits, but their use is quantified in only a handful of studies (e.g. [11]).

To assist with mark-recapture studies, acoustic equipment must allow for real-time detection and localisation of animals that may not be available for visual survey (e.g. animals that spend large proportions of time underwater, or that are at distances beyond the range of visual observations). Acoustic tracking is best suited to animals that vocalise often and loudly, thus blue whales and sperm whales are generally considered good candidates for acoustic studies (e.g. [12], [13]).

Although the main context is mark-recapture, the simulation and the findings have a broader relevance for any endeavour in which encounters with whales needs to be maximised and strict random spatial sampling is not required, for example, deploying telemetry/tracking tags to animals, collecting genetic or biological samples, finding whales for behavioural observation studies etc.).

All known populations of blue whales make loud, low frequency, repeated vocalisations, often in excess of 180 dB re 1 µPa rms @ 1 m [14]. Sperm whales also consistently make repeated vocalisations ([15]), and their echolocation clicks are among the loudest biological sounds, reaching at least 223 dB re 1 µPa peak-to-peak @1 m [13]. These clicks can be detected from many kilometres away using nothing more than a basic hydrophone connected to a speaker or headphones.

The successful use of acoustics in mark-recapture studies will depend not only on the behaviour of the whales, but also on the properties of the acoustic tracking system (e.g. sensitivity, detection range, localisation precision) as well as the properties of the research plaftorm (e.g. vessel speed, time required for tracking and ‘marking’ animals). Various types of instruments have been used from mobile platforms for real-time tracking of marine mammals ranging from hand-held directional hydrophones for tracking sperm whales from small and moderate sized boats (e.g. [8], [11]), towed arrays of closely spaced hydrophones for tracking odontocetes from moderate and large-sized boats (e.g. [6], [16]), and DIFAR (directional) sonobuoys for tracking baleen whales from large boats and/or aircraft (e.g.[17]–[19]). For acoustically-assisted mark-recapture, localisation information may be as simple as a bearing to the sound source, rather than a 2D/3D geo-location, since the main purpose is to track down the animal rather than measure the distance to it, as is required in line-transect surveys [20].

The motivation for the work in this paper is to provide a general framework to assist in planning mark-recapture surveys for whales. We model the process of acoustically-assisted mark-recapture surveys via an individual-based simulation model of animals and survey vessel, to quantify how underlying physical, biological, and logistical factors affect encounter rate.

First, we determine whether the simulation framework provides a reasonable approximation to the real world by simulating a well-studied population of sperm whales from Kaikoura, New Zealand. In this simulation, most of the required parameters have been empirically measured. Hence, this example provides a good test of the validity of the simulation framework.

Second, we apply this framework to a research programme that aims to estimate circumpolar abundance of Antarctic blue whales via a mark-recapture sampling approach. We estimate and compare the encounter rates of both acoustic and visual-only survey methods. Then we investigate the sensitivity of encounter rate to the passive acoustic instrumentation, survey protocols, and whale distribution and behaviour.

## Method

### Simulation Framework

The simulation is an individual-based discrete-time model implemented in R [21], and the code is available as a package (sourceforge.net/projects/watspackage; see Appendix S1). The main purpose of the simulation is to determine the expected number of animals encountered and subsequently marked (in a mark-recapture context) in a typical survey. A summary of the main functions that comprise the simulation is given in Appendix S1. Certain parameters can be set directly in the function calls, and others are set in a parameter file. The simulation can be monitored with various plots (see Appendix S1 for full details). The simulation consists of three components: whales, acoustic tracking system and vessel. Together, these three components provide the framework to simulate a survey.

An acoustic encounter is denoted by the acoustic detection, tracking and subsequent visual detection of an animal. Re-encounters within a season of the same animal are not used. Encounters in an acoustically-assisted mark-recapture study arise from two sources: 1) whales that are tracked acoustically; and 2) whales that are not detected acoustically, but encountered visually, by chance. By simply counting the two types of encounters, the simulation can show the contribution of each to the overall encounter rate.

In addition to an acoustically-assisted mark-recapture survey, a visual-only mark-recapture survey and a visual-only line-transect survey can be simulated. A visual-only mark-recapture survey can be thought of as a baseline case of an acoustic mark-recapture survey, while line-transect surveys are commonly used to estimate abundance, thus forming a basis for comparison.

A simulated survey can be run over a specified time period, area and whale density, with parameters set to match a particular study/application. Each of the components is driven by a set of parameters and decision rules, summarised as follows:

#### Whales

Given that knowledge regarding whale biology and behaviour is usually limited, we have kept the whale model reasonably simple. The main parameters regarding whales are the number and distribution of whales in the study area, vocalisation/dive behaviour, and movement characteristics.

**Figure 1:**
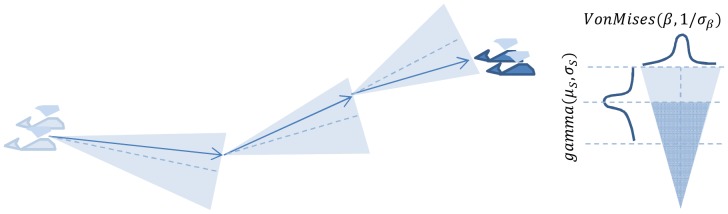
Representation of the correlated random walk used to simulate whale group movement. At each time step, a random variation is added to the cumulative direction (and the distance travelled), causing heading to change smoothly through time.

Our simulation unit was groups of whales rather than individuals. For simplicity, we consider group movements as random, but constrained as a correlated random walk [22], in which each group was assigned a heading that incrementally changed by a random amount at each time step (Figure 1). When groups reach the survey boundary they reverse direction turning back into the survey area.

Often little is known about interactions and movements of whale groups, and in most biological systems groups are not distributed uniformly in space, but rather the distribution is clumped. The mechanism for clumping could be whale-driven (e.g. some social association between groups) and the clump of groups move together, and/or resource-driven (i.e. prey are patchily distributed so whale groups will independently move to these patches). The simulation currently allows for two whale-driven movement models: ‘Clumped’ in which clumps of groups move as a block and ‘herd’ where groups are members of a loosely associated herd and move using a model based on [23], see Appendix S2 for detail.

At its simplest, the simulation can assume all groups vocalise and do so continuously throughout the survey. However, it is also possible to simulate a situation where only a certain proportion of the groups in the survey are vocalising; thus the remaining groups can only be found via visual observations (Appendix S3). If more information on vocalisation rate is known (e.g. temporal variation during diving as per sperm whales) this can also be included. It should be noted, that simulation is at the group level; while individuals may not be vocalising, the group unit may contain another animal that is. In case of applications with very large group size, this would approach equivalence to all animals continuously vocalising.

#### Acoustic tracking system

The main parameters regarding the acoustic tracking system are the frequency of occurrence of listening stations, the effective range of acoustic detection, and precision of acoustic bearings. The effective range of acoustic detection is analogous to effective strip half-width in distance sampling (*sensu* [1]). We consider two types of acoustic instrumentation: hand-held directional hydrophones and DIFAR sonobuoys. The general approach to acoustic tracking is the same for both of these methods: listening stations are conducted at regular intervals, and bearings (with some measurement error) are obtained to vocalising whales within the effective range of acoustic detection.

For hand-held hydrophones, the vessel must stop for a listening station, and typically only a single bearing estimate is obtained for each whale. For DIFAR sonobuoys, the vessel does not need to stop and can continue to follow bearings until the whale is sighted, the sonobuoy expires, or the vessel passes beyond the VHF reception range of the active sonobuoy (i.e. sonobuoys are not retrieved). Furthermore, concurrently monitored sonobuoys deployed several kilometres apart may be used to triangulate the location of the calling animal.

#### Vessel

The main parameters for the simulation of the vessel and team are: the vessel speed; effective half-strip width; g_0_ of visual observers (*sensu* [1]); the time taken to collect animal identifications (photographically or via biopsy); environmental conditions; and, additionally, the duration of the survey.

**Figure 2:**
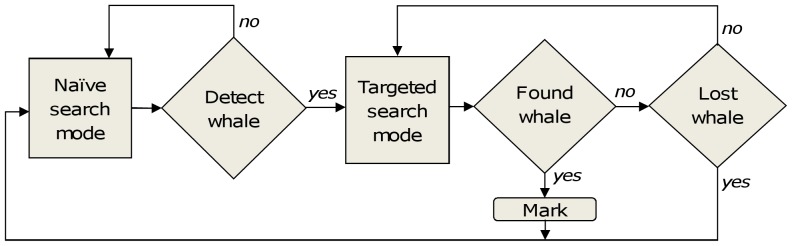
Schematic of simulated vessel tracking modes. The vessel begins in naïve search mode. Upon detecting a group, the vessel switches to targeted mode and, if the whale is found, it proceeds to mark the group; if it is lost it returns to naïve search.

**Figure 3:**
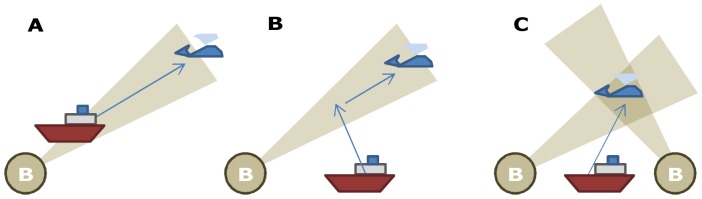
Model for vessel movement given a bearing to a group. Given a bearing there are three vessel actions: (A) if only a single sonobuoy is providing a bearing, and the vessel is close to the buoy-whale bearing line, the vessel follows the bearing; (B) if the vessel is too far from the bearing line, the vessel moves toward the buoy-whale bearing line; (C) If multiple sonobuoys provide bearings, a cross-bearing is calculated and the vessel goes into ‘Direct track mode’ heading straight to the position where the bearings cross (See Table S1).

Listening stations are deployed and the simulated vessel moves around the survey area based on a number of decision rules. The vessel has two main modes: naïve search and targeted search (see Figure 2). When no bearing to a group has been received and no whale is being tracked, the vessel works in naïve search mode. In this mode the vessel moves according to a systematic search, heading to areas which have not been previously searched. Upon acoustic detection of a group of whales, the vessel switches to targeted mode and the vessel moves toward the group based on the bearing information. In targeted mode when using sonobuoys, the direction of travel will depend on the vessel’s location relative to the sonobuoy, as well as the number of active sonobuoys. Specifically, if the vessel is not on the same bearing as the whale it must move to the bearing line (see Figure 3, and Table S1).

Upon encountering a group of whales, time spent collecting identification data from individuals within that group is simulated. This is intended to simulate the collection of photographs and/or biopsy samples, and is based on an average time to “mark”. The probability of successfully obtaining the photograph or biopsy from the animal is not considered in this study. After concluding an encounter, the vessel either returns to naïve search mode, or, if whales were detected previously but not tracked, the vessel heads back to where these whales were detected.

To replicate the effect of overnight tracking (i.e. when the visual search team is not able to operate), the vessel was allowed to listen to and track whales overnight in order to be near whales when visual observation could commence. In the simulations, the operation of the visual team can be linked to weather conditions (that can be generated randomly or sampled from real historical weather observations).

In practice, tracking is sometimes aborted. This can be due to a range of reasons: dangerous ice conditions, whales cease to vocalise, crew becoming disheartened and giving up, or other undetermined practical reasons. To incorporate this we include a probability of abandonment per hour, and, at each time step, the simulation randomly decides if the tracking will be abandoned. Hence, the longer a group is tracked, the greater the probability of its tracking being aborted. Failures due to whales ceasing to vocalise can be incorporated within this parameter or explicitly in the whale vocalising parameters.

### Validation: New Zealand Sperm Whales

To validate the simulation framework, we applied it to a well-studied group of sperm whales. The Otago Marine Mammal Research Group has conducted acoustically-assisted mark-recapture studies of sperm whales in Kaikoura, New Zealand for an average of 6–12 weeks each year between 1990 and 2009. Research protocols have remained remarkably similar throughout the 20 years of research. The work has photo-ID and acoustic tracking as cornerstone research methods, and has addressed a wide range of research questions. The depth and breadth of the research on sperm whales in Kaikoura provides measurements of nearly all of the parameters used by the simulation (see Table 1). The relative constancy of this programme’s protocols, and long time-series of data, provides an ideal dataset for validating the performance of the simulation framework.

**Table 1:** Parameters for the validation study on sperm whales in Kaikoura, New Zealand.

Parameter	Value	Comment/Reference
**Whales**		
Number of whales in survey region+SD	13.8 (1.3)	Table 1 from [31]
Probability of group vocalising	1	Typically all male [32]
Average whale swim speed (km/h)	5.04	[28]
Whale distribution and movement	Uniform/clumped	–
Dive time (min) mean, standard deviation	41.3, 7	[24]
Surface time (min) mean, standard deviation	9.1, 2.5	[24]
Vocalising time (min)	All but last 20% of dive	[33] [15]
Silent time (min)	Time at surface+last 20% of dive	[33] [15]
Bearings obtained per hour	N/A	–
**Acoustics (hand-held hydrophone)**		
Effective acoustic detection range (km) (Note: ESW/2, so the radius )	5.556	Derived from 26 experiments on range of directional hydrophones (unpublished)
Bearing error (std. deviation degrees)	28.7	unpublished data
Distance estimation error (std. deviation)	0.49	unpublished data
Hydrophone dips, distance between (km)	3.6	From range trial (unpublished)
Dwell time (minutes)	2	unpublished data
Amount of time before a lost whale is given up on (h)	2	
**Vessel**		
Simulation length/time step (h)	7.033	Length of a typical day of surveying
Vessel Speed (km/h)	37	From GPS track data
Visual observer ESW/2 (km)	2.0	Based on experience and some incidental sighting data
Visual observer *g*_0_	1	
Time to mark (h)	Varied	Until final dive and fluke up
Maximum tracking time (h)	4	Not really used

The data used for validation consist of 14 seasons, covering 280 days (1844 hours on-effort), between 1995 and 2008. Almost all research was conducted from small outboard-powered boats using a handheld directional hydrophone within a rectangular study area 10×20 nmi south of Kaikoura Peninsula.

Sperm whales in Kaikoura typically spend 30–50 minutes underwater followed by 7–14 min at the surface with some small, but statistically significant, seasonal differences [24]. In Kaikoura, sperm whales make loud echolocation clicks around 80% of time they are underwater, but are usually silent for the last 3–5 minutes of a dive before surfacing and typically remain silent while on the surface [15]. This behaviour was simulated, by using a small time step and restricting acoustic tracking to appropriate periods in the dive cycle.

Ten thousand survey days were simulated using parameters given in Table 1, and simulated encounter rates were compared (in terms of magnitude) to the 14 real-world survey season encounter rates. Since the sperm whale surveys were not conducted in extreme weather conditions, weather was not included in the sperm whale simulation. From the real data it is clear that whales were non-randomly distributed within the survey area. This was replicated in the simulation by using a very approximate polygon based on the data to define a region where whales were present. Furthermore, [8] indicate that the assumption of a uniform distribution is unlikely to hold, so we include a simulation with a clumped distribution of groups (Appendix S2).

### Prediction: Antarctic Blue Whales

Despite being the largest animal known to have existed, relatively little is known about Antarctic blue whales (henceforth blue whale). This makes planning a survey programme difficult; especially when considering novel methods (e.g. acoustically-assisted mark-recapture). The simulation-based approach provides a useful means to explore such methods despite the lack of quantitative information on blue whales. Simulation parameters (Table 2) were derived from several sources, including publications on various blue whale populations, and from acoustic tracking studies that have employed similar methods.

**Table 2:** Parameters for the simulations and sensitivity analysis of Antarctic Blue whales.

Parameter	Value	Comment/Reference
**Whales**		
Density of whales in study area (km^−2^)	0.000539957	Extrapolated from SOWER (Appendix S4)
Probability of group vocalising	0.6	[26], [34], [35] (Appendix S3)
Average whale swim speed (km/h)	4.5	Based on range in [36]
Whale distribution	Random/clumped	Based on examination of Antarctic survey data
Dive time (mean, std. deviation)	N/A	Vocalisation generalised and assumed if an group is vocalising it will not stop during the tracking
Surface time (mean, std. deviation)	N/A	
Singing time (mean, std. deviation)	N/A	
Silent time (mean, std. deviation)	N/A	
Bearings obtained (per hour)	6	Based on empirical data
Clumping		See Appendix S2
**Acoustics (DIFAR Sonobuoys)**		
Effective acoustic detection range (km)(Note: ESW/2 so the radius )	50	Preliminary analysis of unpublished data [29]
Maximum sea state acoustics operate	5	Experience from Ant. survey
Buoy transmission time (h)	8	Based on empirical data from Antarctic survey
Buoy VHF range (km)	18.52	
Bearing error (std. deviation degrees)	15	
Bouy drop rate, searching/targeting (h)	4/1	Based on empirical data
Amount of time before a lost whale is given up on (h)	3	After this time if another group is detected targeting is changed
Maximum tracking time (h)	14	After this time the vessel switches to naïve search mode
**Vessel**		
Simulation length/time step (h)	240/0.5	10 days of voyage
Vessel Speed (km/h)	20.3	SOWER vessel
Visual observer ESW/2 (km)	3.5 km	SOWER [25]
Visual observer *g*_0_	1	SOWER
Lowest sightability visual team operates	2	As per SOWER
Dawn and Dusk	6 am and6 pm	Based on typical workday
Probability of abandonment	0.02	Based on Antarctic survey
Time to mark (h)	1.51	SOWER surveys ([37] and references therein). This aligned with experience on the Antarctic pilot survey.

The IDCR/SOWER surveys (International Whaling Commission circumpolar surveys; henceforth SOWER), in particular blue whale studies, provide useful information about density of blue whales in the Southern Ocean ([25]; Appendix S4). Acoustic studies conducted during the SOWER cruises also allowed for quantification of the vocalisations of blue whales [26]. In addition to describing call properties, [26] also found 14 out of the 15 recorded groups of blue whales were vocalising.

Empirical measurements of the detection range of blue whales have been made in a handful of studies using a variety of different instrumentation [12], [27]. These studies report detection distances from 20 to 200 km, although none of these studies were conducted in real-time. Sources of variability in detection range include: instrumentation, bathymetry, oceanographic conditions, and source level of vocalisations. Only one survey of Antarctic blue whales has used real-time acoustic tracking as a primary means for locating whales [28]. Instead of introducing additional parameters for which we had little data (e.g. propagation loss coefficient, surface duct height, source level of whales, directivity of whales and receivers), we opted to use empirical evidence from prior studies in the Antarctic and Subantarctic to inform our initial estimates of detection range. Based on this limited information, an effective range of 50 km was used. This value concurs with a preliminary analysis of unpublished data [29]. We then conducted a sensitivity analysis to see how, on average, detection range would affect the encounter rate.

Based on a recent Antarctic survey [28], we assumed that whales could be acoustically tracked 24 hours a day, including overnight (between 6 pm and 6 am, local time) and during all but the most severe weather. To quantify the advantage gained from the ability of acoustics to operate overnight we repeated the simulation limiting acoustics to operate during daylight hours.

To include the effect of weather, we sampled weather observations from the SOWER dataset. During bad sighting conditions visual surveys were suspended, but acoustic tracking continued. When the sea state was greater than Beaufort 5, all operations were suspended.

One thousand surveys of a 10 day length were simulated (parameters used are given in Table 2), and the encounter rates examined. Encounter rates correspond to mean encounters per hour over the full survey, including day/night, and off-effort times due to weather. The simulation served as further validation and facilitated comparison of survey designs.

### Sensitivity Study: Antarctic Blue Whales

Given the uncertainty of many of the parameters in the Antarctic blue whale study, we conducted a sensitivity study to examine the sensitivity of encounter rate to density as well as various other parameters. From the SOWER data and the recent Antarctic blue whale survey, distribution of groups appears to be non-uniform. Therefore, we explored the effects of non-uniform distribution of groups on encounter rate by incorporating group associations (see Appendix S2) and comparing this to a simulation with a uniform distribution of groups. The other parameters investigated in the sensitivity study were:

- effective acoustic detection range (10, 25, 50 and 100 km);
- proportion of whale groups vocalising (25, 50, 75 and 100%);
- whale swim speed (0.1, 4.5, 15 and 30 km/hr);
- sonobuoy VHF transmission range (10, 18.5 and 30 km) and
- the acoustic bearing error (10, 20, 30 and 40°).

To determine the sensitivity of encounter rate to each parameter, 1000 surveys were simulated for each value of each parameter over a range of plausible densities, while values for other parameters were set to those in Table 2.

## Results

### Validation

**Figure 4:**
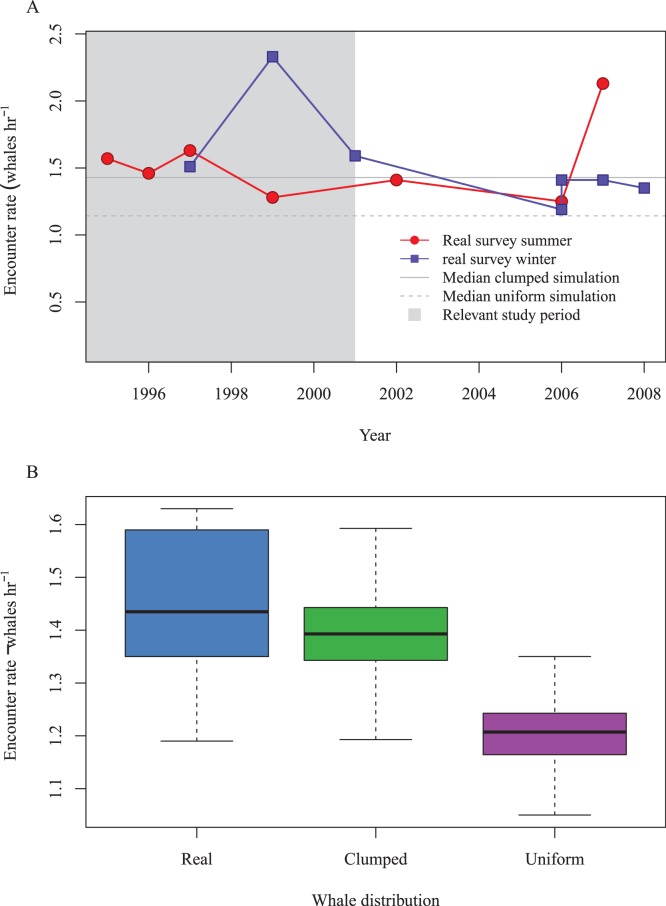
Results from simulation of Kaikoura sperm whales. (A) Encounter rates of sperm whales in Kaikoura observed in the summer (red line) and winter (blue line). The solid horizontal line denotes the median simulated encounter rate for a given clumped whale distribution, and the dashed line, the median of the uniform distributed whale simulation. The solid gray rectangle denotes the period of the data for which the number of resident whales used in the simulation was derived [31]. (B) Comparison of seasonal variance of estimated encounter rate for real data, simulation with clumped and uniform whale distribution.

From the New Zealand sperm whale surveys the actual encounter rates ranged in each season between 1.19 and 2.33 whales per hour, with a median of 1.44 (mean = 1.54) (Figure 4a). Median simulated encounter rate with uniform movement/distribution was 1.14 whales per hour (mean = 1.21). For simulations that incorporated clumping of distribution/movement, median encounter rate increased to 1.43 (mean = 1.40). Variance in encounter rate was underestimated in the situations compared to the real surveys (Figure 4b). Simulations showed a clear benefit of using acoustics; surveys that did so had about four times the average encounter rate of visual-only mark-recapture simulations.

### Prediction

**Figure 5:**
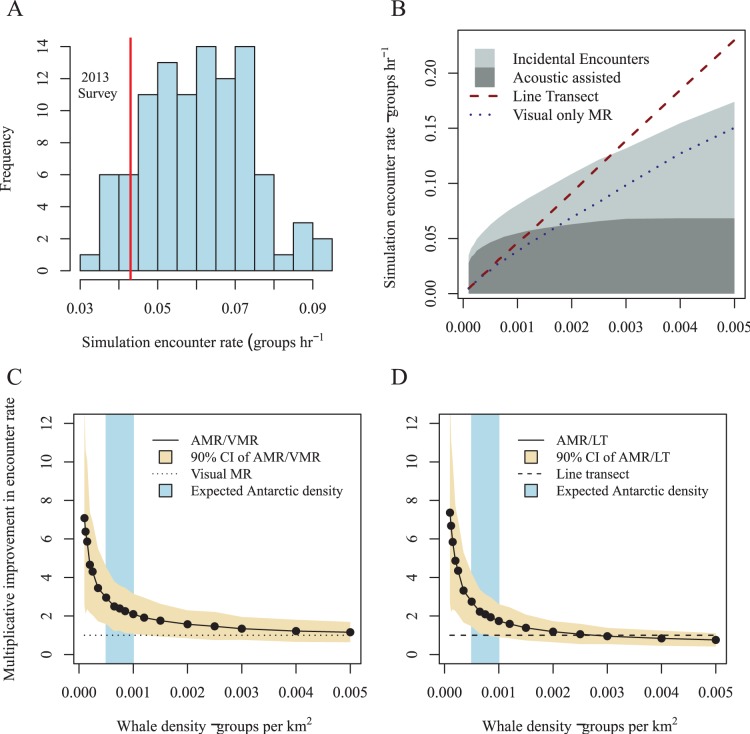
Results from simulation of Antarctic Blue whales. Simulated encounter rates for Antarctic blue whales with clumped whale distribution: (A) at group density predicted for 2013 compared to rate from actual 2013 survey (red line); (B) mean encounter rates for a range of densities. Light shading indicates encounters due to acoustics. Dark shading indicates those from visual observation. The lower panels show the multiplicative improvement of an acoustics-assisted mark-recapture survey (AMR) over (C) a line-transect survey (LT), and (D) a visual-only mark-recapture survey (VMR).

Predicted encounter rate for an Antarctic blue whale survey ranged from 0.03 to 0.09 groups per hour (Figure 5a) over the group densities derived in Appendix S4. The 2013 Antarctic blue whale voyage made use of acoustic tracking and encountered 0.043 groups per hour [29], which lay at the lower end of results from the simulations.

At higher densities, the acoustic tracking component levelled off considerably, due to time spent in tracking and “marking”. The incidental sighting component also levelled due to “marking” time but not to the same extent (Figure 5b).

For a study of blue whales, it is pertinent to compare the performance of the methods at the estimated current population density. Our simulations indicate that the use of acoustics would improve encounter rate by around 2.1– 3.0 times compared to a visual mark-recapture (see Figure 5c) and by around 1.7–2.7 times that of a line-transect survey (Figure 5d).

### Sensitivity Study

**Figure 6:**
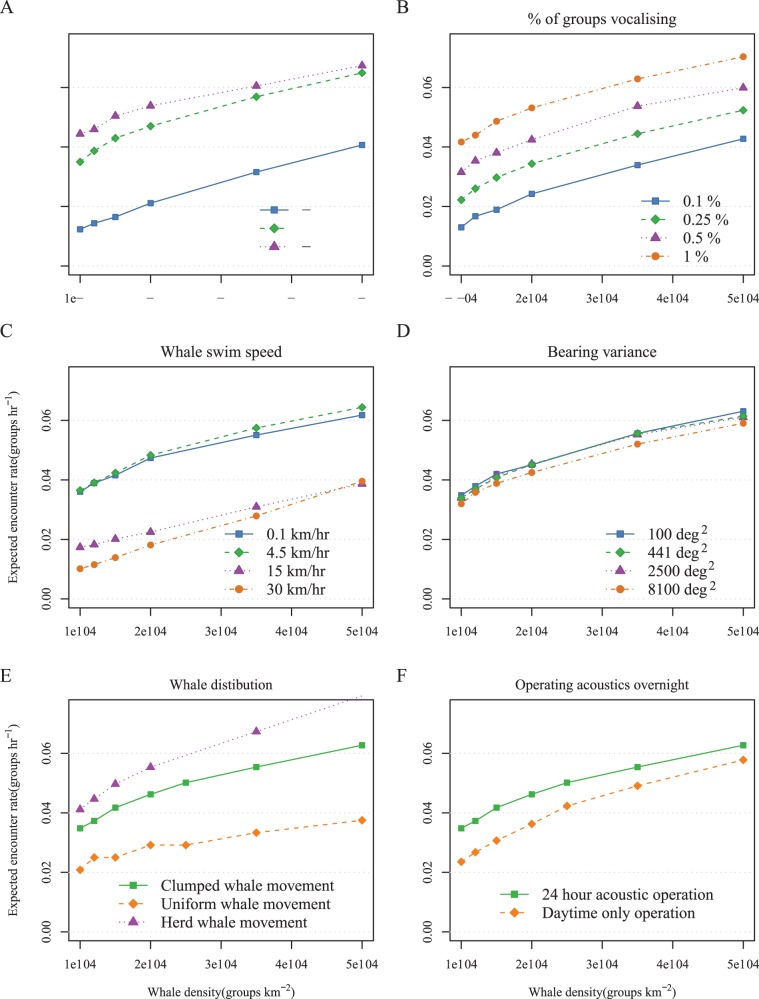
Results from the sensitivity study. Sensitivity study of mean encounter rate to (A) acoustic detection range, (B) percentage of whale groups vocalizing, (C) whale swim speed, (D) sonobuoy VHF range, (E) comparison of acoustically-assisted encounter rate from operating acoustics 24 hours a day versus daylight hours (6 am–6 pm) and (F) the effect of clumped distribution/movement versus uniform distribution of groups.

Encounter rate was most sensitive to effective acoustic detection range. As expected, decreasing effective range gave fewer whale encounters (Figure 6a) and very low values of acoustic range result in encounter rates similar to those from a visual mark-recapture survey. The proportion of whale groups vocalising also had a strong effect on encounter rate (Figure 6b). Whale swim speed affected the result in a non-linear fashion (Figure 6c). Encounter rate was insensitive to all but extreme bearing errors (Figure 6d). The VHF transmission range of sonobuoys (not shown) had minimal effect on encounter rate, but did have an effect on the number of sonobuoys used during a survey (i.e. shorter transmission ranges require more sonobuoys for similar coverage). Simulations of acoustic tracking of clumped distributions yielded encounter rates 49%–72% higher than uniform distributions (see Figure 6e). Simulations of visual-only surveys gave no change in mean encounter rate, but did show greater variance for clumped distributions. The use of acoustics to track animals overnight gave approximately a 9%–48% gain in encounter rate over the range of expected densities of Antarctic blue whales (Figure 6f).

## Discussion

Due to increased detection range, acoustically-assisted mark-recapture was expected to have a distinct advantage over solely visual-based methods when studying blue and sperm whales. The simulation framework quantified this advantage and facilitated exploration of the effect of the main parameters on encounter rate.

### Simulation vs. Reality

There is always a danger that a simulation may fail to capture the principal factors influencing the response of interest. To validate our simulation, we applied it to a large dataset collected from New Zealand sperm whales. The simulation performed well, with mean simulated encounter rates of clumped whales within 10% of the mean of those seen in the real world. Variance in encounter rate was underestimated by the simulation; probably because the simplicity of our model did not fully capture factors present in the real world. Some caution must be taken when comparing the simulation results to the real world encounter rates, whether they closely agree (Figure 4) or there is some discrepancy (Figure 5a). In both cases there are still uncertain parameters that, if changed, could influence the result considerably, e.g. underlying whale distribution and density. Extrapolating the validation to an ocean-wide low density population is not ideal. However, the validation is applicable for the simulation concept and idea of modelling complex surveys, rather than a validation of specific parameters/modelling.

Of necessity, the simulation makes several simplifications and omissions. For example, the decision rules do not capture the intricacies of human decision making. When no whales are detected, a researcher is likely to use other information to decide where to relocate, such as: historical whale sightings/distribution, weather forecasts, potential krill distribution or other environmental data.

The simulation framework does not consider the use of long-range detections, which may have the potential to inform decisions on broad-scale vessel movements. In practice, long-range acoustic detections are only one of many inputs into the decision making process, e.g. other inputs include historical sighting data, weather forecasts, logistics, sea ice conditions, models and understanding of the ecosystem, etc. Hence, it is difficult to quantify the effect of having long-range detections available. One possible way to incorporate limited broad-scale knowledge of whale distribution and location is to simply use an estimate of whale density in the simulation corresponding to high density areas, rather than the overall uniform density (as per Figure 5).

We made additional simplifications regarding whale movement. Simulated behaviours were simplistic because few quantitative data were available, thus simple models were deemed most appropriate. However, more complex models could easily be implemented in scenarios where more information is known. It should also be noted that the simulated encounter rates do not allow recapturing of groups previously found within a season.

Some of the parameters in the simulation are highly uncertain. For example, estimating effective acoustic range is not a trivial exercise as there are complications due to whale movement between initial detection and sighting, and the fact that the range will depend on ambient noise, source level, depth of vocalisation, and propagation characteristics within the water mass [30].

Technically, in our approach to non-uniformity of whale groups, we are compounding non-independent movement with non-uniform distribution. However, there could be a situation in which groups move independently (i.e. do not exhibit clump movement) but the distribution of groups is non-uniform. For example, if independent groups of whales move between, and feed on, localised krill swarms.

### Mark-Recapture Heterogeneity

Abundance estimation via mark-recapture is based on a number of assumptions, which, if invalid, may lead to biased and misleading results. Therefore, it is important to consider how using an acoustic-assisted approach will affect the assumptions of the subsequent mark-recapture analysis.

In many ways, in terms of a mark-recapture analysis, the data resulting from an acoustic-assisted survey will be no different to a regular visual survey. While there is some auto-correlation in sampling space as the vessel moves in a continuous fashion, this will not pose a problem if the recapture sampling unit is chosen correctly. Recaptures should only be considered across discrete independent surveys in time and not within the same survey. For example, in the simulation of Antarctic Blue whales, recaptures within the season are ignored and only recaptures across years/seasons are considered. However, in any particular application, it would be advisable to examine the population for sub-structure/mixing and make sure that the spatial sampling of the non-random acoustic survey does not introduce any sampling bias.

The biggest concern arises around acoustic-assisted sampling being biased toward vocalising animals. If not all animals vocalise, or some vocalise more than others, this will result in unequal likelihood of capture. Such heterogeneity may be reduced due to the nature of grouping of animals (e.g. if vocalising and non-vocalising animals mix at random within groups). Also, it should be noted that if the vocalising behavior of the animals is constant through time, and only a relative index of abundance is required, the heterogeneity will not be as relevant. If an absolute abundance estimate is required, some correction/model will be needed to produce unbiased mark-recapture estimates. This could be accomplished with other independent data on vocalising behavior, detection and sex ratios, etc.

### Prediction and Sensitivity

Simulations of Antarctic blue whales suggest an improvement in encounter rate by using acoustics compared to visual mark-recapture methods. Interestingly, as whale density increases, the improvement from using acoustics diminishes. In the case of extremely high densities, whales could be seen sufficiently often during a visual-only survey that the ability to track them acoustically at long-range offers little advantage.

As expected, the encounter rate in simulated line-transect surveys showed a linear relationship to whale density. In contrast, encounter rates of the acoustically-assisted and visual mark-recapture approaches were not linear with density, decreasing as whale density increased. As density increases a greater proportion of the total time is spent photographing and/or biopsying individuals. Due to this effect, there is a point (around 0.002 whales per km^2^; Figs. 5a, 5b & 6b) at which expected encounter rate from an acoustically-assisted mark-recapture survey becomes lower than that of a visual line-transect survey.

Our simulations revealed acoustically-assisted surveys to be far superior to purely visual surveys in scenarios incorporating clumped distributions, which we suggest is the more realistic case. This appears to be mainly due to the extra search range provided by acoustics. Additionally, the adaptive design of a mark-recapture survey allows the vessel to stay and focus on these areas of higher density rather than continue along a transect, as required by the line-transect approach.

The sensitivity study gave a clear indication of how the main parameters affect encounter rate. Information such as this could be invaluable in decision-making regarding specifications of acoustic systems and vessel protocols when planning a survey. In fact, preliminary versions of the simulation software were used to estimate the number of sonobuoys that might be used during planning stages of the 2013 Antarctic Blue Whale Voyage [29].

Simulations revealed that acoustically assisted mark-recapture surveys yielded consistently higher encounter rates than purely visual mark-recapture surveys over all whale densities. At densities expected in the Southern Ocean, acoustically-assisted mark-recapture also gave a higher encounter rate than line-transect distance sampling. When studying rare species, approaches that increase encounter rates are highly desirable. How these encounter rates affect precision is an interesting question, and with further simulation using a population model, it is possible to estimate this. Furthermore, there are other aspects of the survey methods beyond encounter rates/precision to consider, such as logistical constraints. Such considerations are non-trivial, and are well beyond the scope of this paper.

## Conclusion

This work has demonstrated that simulation-based approaches can provide useful guidance regarding the design of surveys for marine mammals, particularly where there is uncertainty and missing information. The simulation faithfully reproduced average encounter rates for long-running studies of sperm whales and preliminary studies of Antarctic blue whales. Furthermore, this work has helped quantify and disentangle some of the mechanisms and relationships between acoustically-assisted/visual mark-recapture and line-transect approaches. In particular, we have quantified how encounter rates are influenced by changes in population density and the importance of marking time in mark-recapture. Finally, our model suggests that acoustically-assisted tracking has provided a tangible benefit for a study on sperm whales, and would provide a tangible improvement in encounter rate over a visual-based approach for future Antarctic blue whale surveys.

The focus of the simulation has been blue and sperm whales, but the general findings hold for other whale species that can be tracked acoustically and the simulation can be re-applied to any whale species for specific results. Furthermore, the framework and concept could be extended to other complex survey design scenarios that are difficult to accommodate with standard statistical methods, for example, comparing traditional line-transect design to more adaptive searching mark-recapture type surveys.

## Supporting Information

Table S1 **Further information on simulated acoustic tracking vessel decision rules.**
(DOCX)

Appendix S1 **Help for the WATS R package.**
(DOCX)

Appendix S2 **Further detail on movement models.**
(DOCX)

Appendix S3 **Further information on whale vocalisation/dive model.**
(DOCX)

Appendix S4 IDCR/SOWER calculations for Antarctic blue whales.
(DOCX)
