# Supplementary material for: A Simulation Study of Acoustic-Assisted Tracking of Whales for Mark-Recapture Surveys
Source: PLoS One. 2014 May 14;9(5):e95602. doi: 10.1371/journal.pone.0095602 (PMC4020746; doi:10.1371/journal.pone.0095602)
Supplement: Table S1 — Further information on simulated acoustic tracking vessel decision rules. (DOCX) [file pone.0095602.s001.docx]

#### **Table S1**: Further information on simulated acoustic tracking vessel decision rules

| Mode | Description | Comment |
| --- | --- | --- |
| Naïve search mode | When no groups are detected the vessel moves systematically through the survey area, by moving to the neighbouring region where it has surveyed the least | This is slightly less than optimal compared to reality, where the researcher could use other knowledge to inform the decision on where to try, e.g. previous un-tracked detections. |
| Follow Bearing Mode | When a single bearing is obtained and the vessel is close enough to the buoy^6^ it is appropriate to follow the bearing in order to encounter the targeted group. | Noise was incorporated in the bearing. |
| Intercept Bearing Mode  (*Move to track line)* | When a single buoy detects a group but the vessel is deemed to be too far from the buoy to use the bearing^[[1]](#footnote-1)^ the vessel moves closer to a point on the bearing line. | This was replicated as heading to the waypoint the same distance the vessel is from the buoy but along the whale bearing. In reality, another buoy would likely be deployed to obtain cross bearings. |
| Direct Track Mode  *(Follow Cross bearing)* | When 2 or more buoys detect a whale the vessel moves toward the cross bearing | Bearing variance was propagated thru to the cross bearing |

1. The vessel is deemed close enough to the buoy to follow the bearing directly if it is within x km of the buoy and the buoy bearing difference between the whale and the vessel is less than ε, where e is given by the maximum angle, that would put the bearing line within visual (ESW) range at the next time step, that is $\varepsilon=\tan^{-1}\left(ESW/2\right)\times vesselspeed\times timestep$ [↑](#footnote-ref-1)
