# Supplementary material for: A Simulation Study of Acoustic-Assisted Tracking of Whales for Mark-Recapture Surveys
Source: PLoS One. 2014 May 14;9(5):e95602. doi: 10.1371/journal.pone.0095602 (PMC4020746; doi:10.1371/journal.pone.0095602)
Supplement: Appendix S1 — Help for the WATS R package. (DOCX) [file pone.0095602.s002.docx]

## **Appendix S1:** The WATS package

### Introduction

The R package WATS (Whale Acoustic Tracking Simulator) is available from

[sourceforge.net/projects/watspackage/](https://sourceforge.net/projects/watspackage/)

It requires ≥R2.15.*. Its main purpose is to run an individual-based simulation of an acoustically-assisted mark-recapture survey of whales. Once the package is loaded in R, help can be found typing ?WATS.

library(WATS)

?WATS

The three main functions are:

- ***fn.simulate.survey*** - runs a single individual-based simulation of an acoustically-assisted survey. This can be useful to check the simulation is working, and examine how the various components are interacting.
- ***fn.simulation^[[1]](#footnote-1)^*** – is a wrapper around *fn.simulate.survey ,*in order to run multiple replicate surveys that can be used to produce expected encounter rates and variance (as used in Fig. 5 in the main paper).
- ***fn.simulation.sensitivity^1^*** – is a wrapper around *fn.simulate.survey,* to test sensitivity of certain parameters (as used to produce Fig. 5, 6, 7 of the main paper).

### Running a simulation

There are two ways to set the various parameters in the simulation:

1. Modifying the parameter text files – Almost all parameters (see Table 1 and 2 of main paper) are read in from a text file, which is stored in the same directory as the R workspace. The file name is specified when calling the simulation function with the parafile argument. To begin, you can create a default parameter file using the function *fn.io.make.parafile*.
2. For quick temporary changes, some parameters can be set via the parameter argument in the function call.

So, to run a single simulations use the function *fn.simulate.survey*. For example:

fn.io.make.parafile('AntarcticBlue_para.txt', type = 'AntBlue')
# Then edit the fike 'AntarcticBlue_para.txt’ as required

ansAntBlue <- fn.simulate.survey(WhaleDensity = 0.0005, Eff_hr = 240,
surveytype = 'Largescale', parafile = 'AntarcticBlue_para.txt',
simpara = list(Tstephr = .5))

runs a suite of plots to monitor the progress of the simulation are displayed (Fig. S1.1 and S1.2).


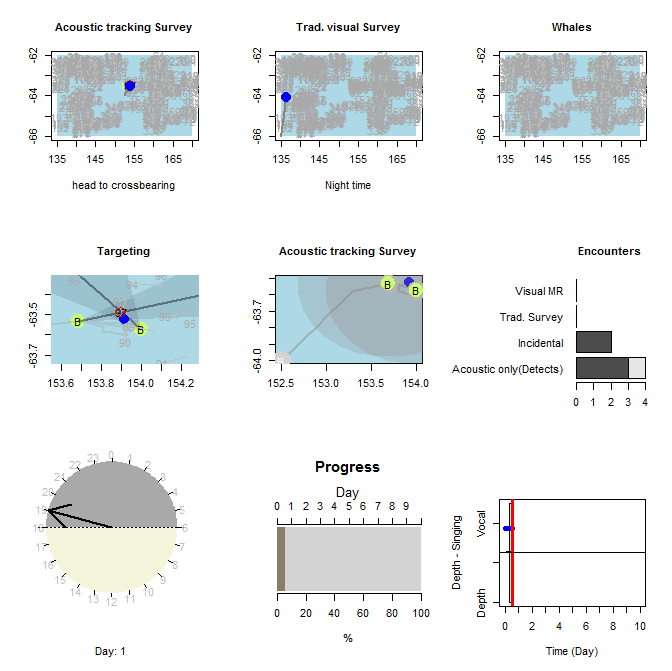


#### **Fig S1.1:** Example of plots to monitor the simulation

**Important Note**: the plotting process can be slow, so the simulation runs much quicker with fewer plots or with no plots at all. (The simulation plots can be replayed later via the function *plot.sim_replay*. To specify what plots are shown during the simulation, set plottype in the control argument.)

fn.io.make.parafile('NZspermwhale_para.txt' ,type = 'NZsperm')

ansNZ <- fn.simulate.survey(Nwhales = 14, Eff_hrs = 7, surveytype = 'Smallscale', parafile = 'NZspermwhale_para.txt', **control=list( plottype = c(1, 2) ),** simpara = list(Tstephr = 2/60) )

plot.sim_replay(ansNZ, plots = c(6,7))


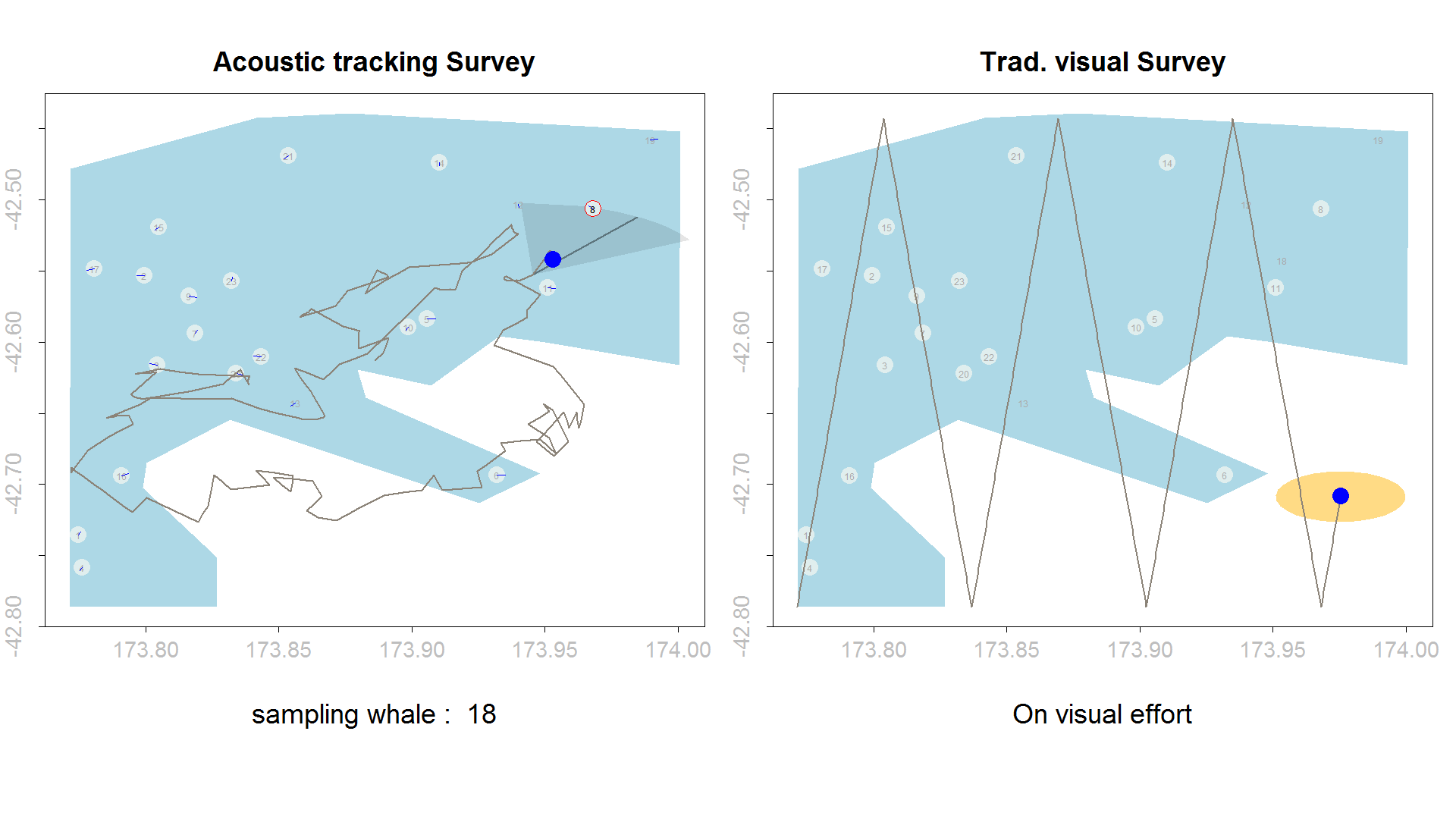


#### **Fig S1.2**: Example of a simulated sperm whale survey

The function *plot.sim_replay* can be used to replay the specified progress simulation plots for a given simulation object. Thus, the user can run the simulation without plots and then revisit it looking at a specified plot.

### Simulation of replicate surveys

To simulate replicate surveys, use the function *fn.simulation*. The arguments are similar to *fn.simulate.survey* except it also includes an argument called n which is the number of replicate surveys. For example:

anssimAntBlue < -fn.simulation(n = 100, WhaleDensity = 0.000539957, Eff_hr = 240, surveytype = 'Largescale', parafile = 'AntarcticBlue_para.txt', simpara = list(Tstephr = 0.5) )

### Sensitivity Simulation

To investigate the sensitivity of certain parameters use the function *fn.simulation.sensitivity*. Simply specify the parameter (see function help for list) and the values to test. For example:

sensDetect<- fn.simulation.sensitivity(values = c(15, 50, 100),
Density = c(0.00010, 0.00012, 0.00015, 0.00020, 0.00035, 0.00050), surveytype = "Largescale", parafile = "AntarcticBlue_para.txt", n = 1000, Eff_hrs = 240)

# If you are on a multi-core Linux machine run the parallel version.

sensDetect<- fn.simulation.sensitivityP(values = c(15, 50, 100),
Density = c(0.00010, 0.00012, 0.00015, 0.00020, 0.00035, 0.00050), surveytype = "Largescale", parafile = "AntarcticBlue_para.txt", n = 1000, Eff_hrs = 240, mc.cores = 18)

### Viewing Results

The user can view the objects returned by the simulations (see function help files in R for more information). The output of most simulation functions contain expected encounter rates, either in the main returned object, or in the list elements ‘stats’ or ‘summary’. For example,

**EA** = From an acoustically-assisted mark-recapture survey whale groups per hour
**ESp** = The contribution to EA from incidental visual sightings
**ES** = The encounter rate of a visual line-transect survey
**EVMR** = The encounter rate from a visual mark-recapture survey

Also, there are a number of functions to plot the results of the simulations:

- ***plot.summary*** - plots the summary of encounter rates from a simulated survey object created by *fn.simulate.survey*.
- ***plot.diagnostics*** – plots some auxiliary information such as track length/time from the simobject resulting from *fn.simulate.survey*.
- ***plot.surveymap*** – for interest, this is a plot showing a map of the data that would result from the simulated survey. This plot is useful to compare to any available real survey data to get a subjective sense of whether the clumping of the whale distribution was sensible.
- ***plot.simulation.sensitivity* –** plots the result from *fn.simulation.sensitivity* as per Fig.7 of the paper.

For example:

library(WATS)

# Simulation

anssimAntBlue <- fn.simulation(n = 100, WhaleDensity = 0.000539957, Eff_hr = 240, surveytype = 'Largescale', parafile = 'AntarcticBlue_para.txt', simpara = list(Tstephr = 0.5) )

# Plot diagnostics

plot.diagnostics(diagnostics = anssimAntBlue$diagnostics)

# Plot summary

plot.summary(anssimAntBlue)

# Sensitivity

sensDetect<- fn.simulation.sensitivity(values = c(15,50,100),
Density = c(0.00010, 0.00012, 0.00015, 0.00020, 0.00035, 0.00050), surveytype = "Largescale", parafile = "AntarcticBlue_para_clump.txt", n = 1000, Eff_hrs = 240, userweather = sower.weather, mc.cores = 18)

sensDetect<- fn.simulation.sensitivity(values = c(15,50,100),
Density = c(0.00010, 0.00012, 0.00015, 0.00020, 0.00035, 0.00050), surveytype = "Largescale", parafile = "AntarcticBlue_para_clump.txt", n = 1000, Eff_hrs = 240, userweather = sower.weather, mc.cores = 18)

# The plot the result

plot.simulation.sensitivity(sensDetect)

### Useful Information

#### **Table S1** : Summary list of functions

| **Function** | **Default** |
| --- | --- |
| fn.io.make.parafile | function (parafile = "SimParameters.txt", type = "NZsperm", overwrite = F) |
| fn.simulate.survey | function (Eff_hrs = 128, Nwhales = NULL, WhaleDensity = NULL,  surveytype = "Largescale", parafile = NULL, userweather = NULL,  region = NULL, simpara = list(), control = list(), workpara = list(),  cols = list()) |
| fn.simulation | function (n = 10, Nwhales = NULL, Eff_hrs = 7, WhaleDensity = NULL, silent = T, progressbar = T, surveytype = "smallscale", parafile = NULL, savediagnostics = T, control = list(), ...) |
| fn.simulationP | function (n = 10, Nwhales = NULL, Eff_hrs = 7, WhaleDensity = NULL, silent = T, surveytype = "smallscale", parafile = NULL, savediagnostics = T, control = list(), mc.cores = 24, ...) |
| fn.simulation.sensitivity | function (parameter = "ADetectRange", values = c(10, 20, 30),  Density = c(0.005, 0.01, 0.02, 0.03, 0.04), n = 3, Eff_hrs = 128,  surveytype = "Smallscale", parafile = "NZspermwhale_input.txt",  simpara = list(), ...) |
| fn.simulation.sensitivityP | function (parameter = "ADetectRange", values = c(10, 20, 30),  Density = c(0.005, 0.01, 0.02, 0.03, 0.04), n = 3, Eff_hrs = 128,  surveytype = "Smallscale", parafile = "NZspermwhale_input.txt",  simpara = list(), control = list(), mc.cores = 24, userweather = NULL,  ...) |
| plot.diagnostics | function (simobj) |
| plot.simulation.sensitivity | function (ans = NULL, xlab = "Density of whale groups (per km2)",  ylab = "Expected encounter rate", main = NULL, addlegend = T,  ltys = c(1, 2, 3, 4, 5, 6), pchs = c(22, 23, 24, 19, 25,  21), legendtitle = NULL, legendxy = NULL, xaxt = "s",  yaxt = "s", ylim = NULL, cols = c("black", "black", "black",  "black", "black", "black"), bg = c("gray", "gray", "gray",  "gray", "gray"), lineunits = "", addSightSurvey = T,  remote = NULL, unit = 1, ...) |
| plot.summary | function (simobj) |
| plot.surveymap | function (ans, cols = list()) |

#### **Table S2** : Default objects used to control simulation from function calls

| **Object** | **Defaults** |
| --- | --- |
| **Control** | trace = NULL, freeaspect = T, Horizaspect = F, pause = F, zoom = 40, smallfootprint = F, plottype = c(1, 2, 3, 4, 5, 6, 7, 8, 9), singleplotwindow = T, rand.seed = NULL, Animate = F |
| **Para** | Tstephr = 0.5, ignoreNight = F, AcousticsStopNight = F, stopbadweather = F, ADetectRange = NULL, bouyUHFRange = NULL, dwelltime = NULL, move = NULL, PS = NULL, WS = NULL, PrSc = NULL, BearingError = NULL |
| **Workpara** | ngridLon = NULL, ngridLat = NULL |

#### **Table S3** : Simulation monitoring plot types

| **ID** | **Textname** | **Example** |  | **ID** | **Textname** | **Example** |
| --- | --- | --- | --- | --- | --- | --- |
| **1** | **"acoustic"**  *View the acoustic Mark-recapture survey* | 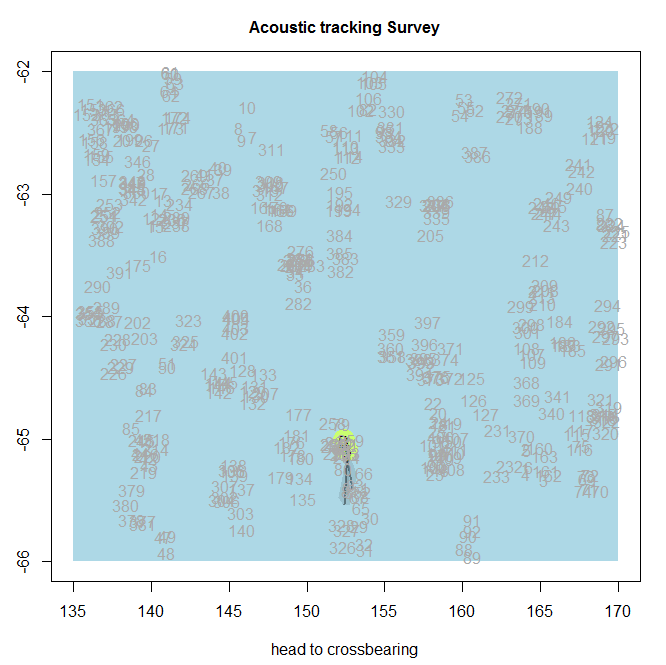 |  | **6** | **"tally"**  *A running tally of the encounter rate for each survey method* | 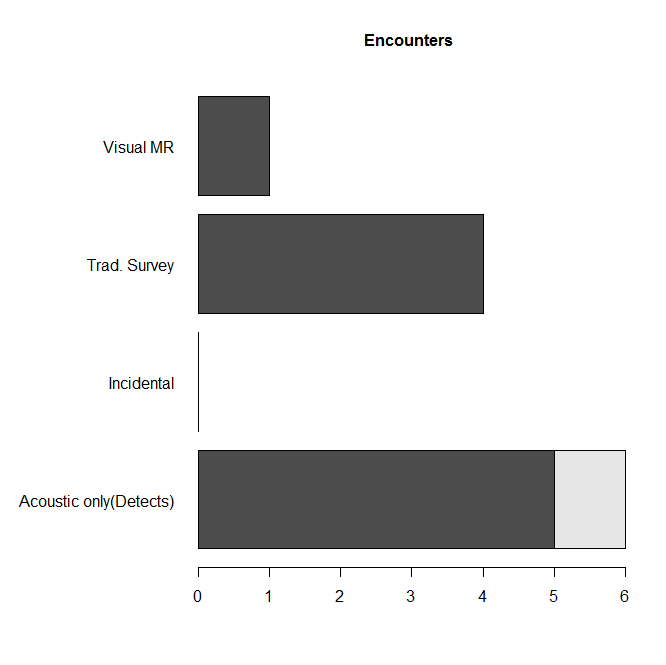 |
| **2** | **“visualLT"**  *View the visual line transect survey* | 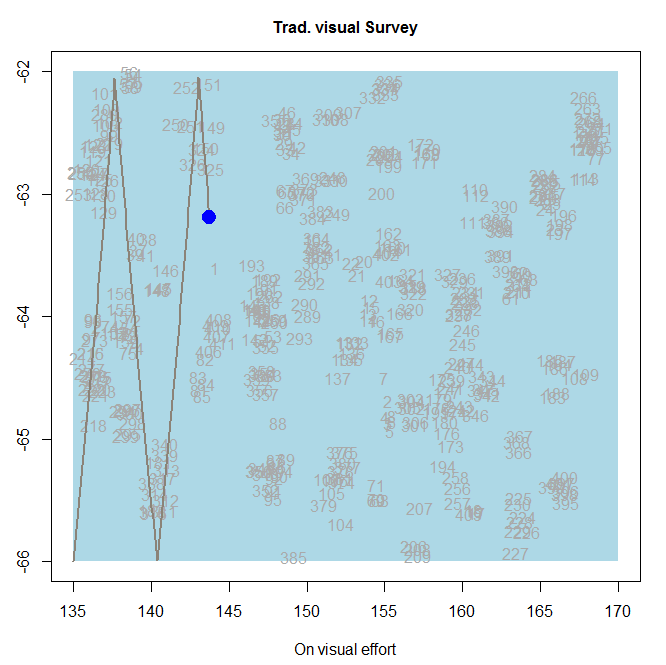 |  | **7** | **“clock”**  *Time of day* | 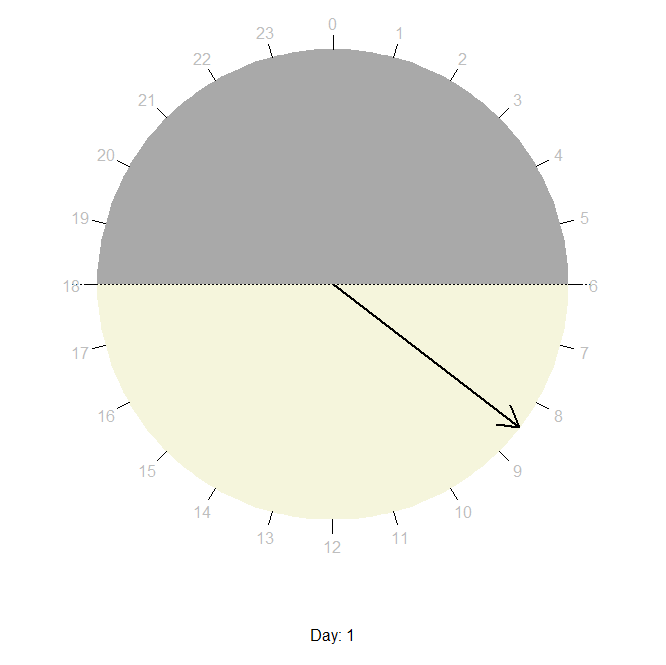 |
| **3** | **"whales"**  *Plot of whale group movement* | 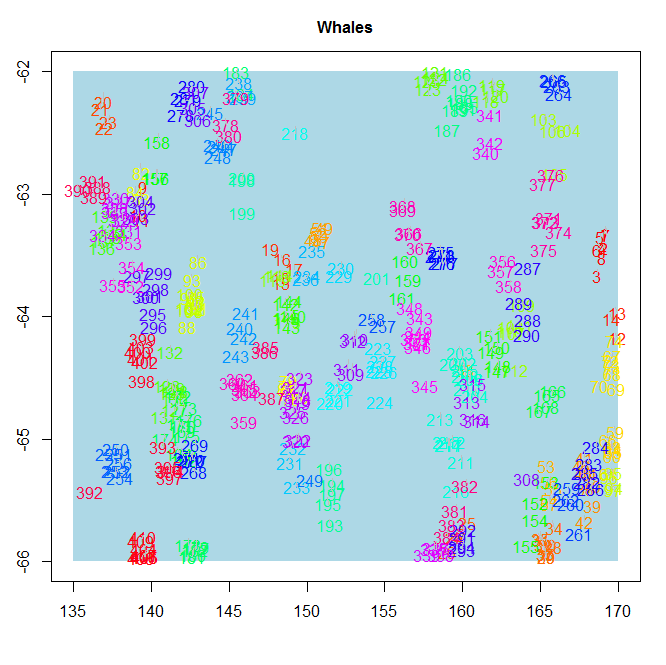 |  | **8** | **“progress”**  *Progress bar* | 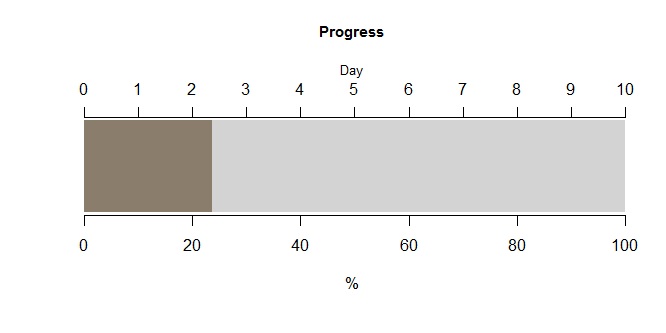 |
|  |  |  |  | **9** | **"dive"**  *Dive/vocal of tracked group* | 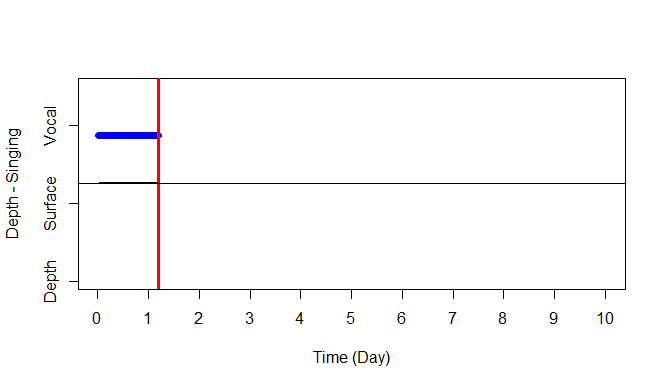 |
| **4** | **"target"**  *Acoustic targeting of whale group* | 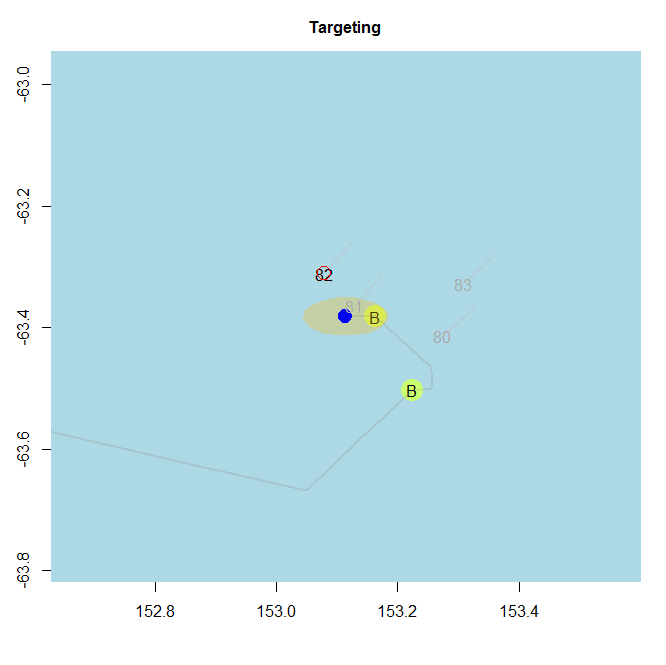 |  | **10** | **"weather"**  *A plot of weather i.e. sightability and seastate* | 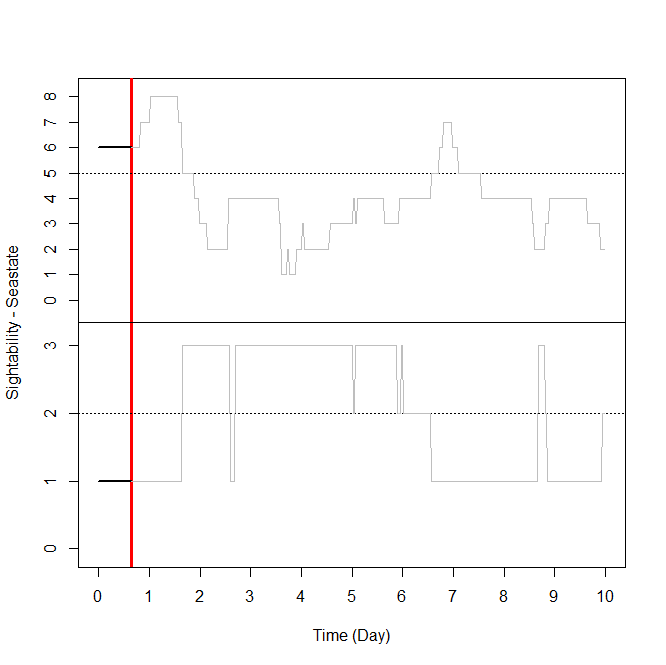 |
| **5** | **"bouys"**  *All sonobouys dropped so far* | 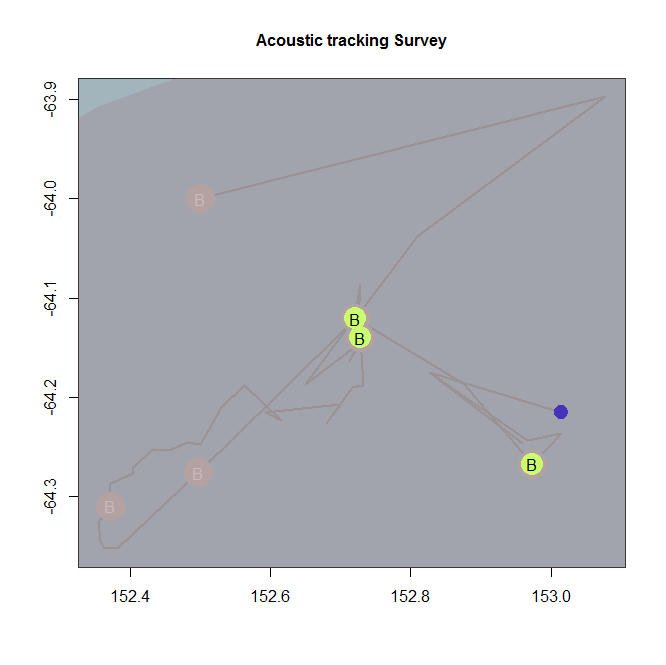 |  | **12** | **"visualMR"**  *View the visual mark-recapture survey* | 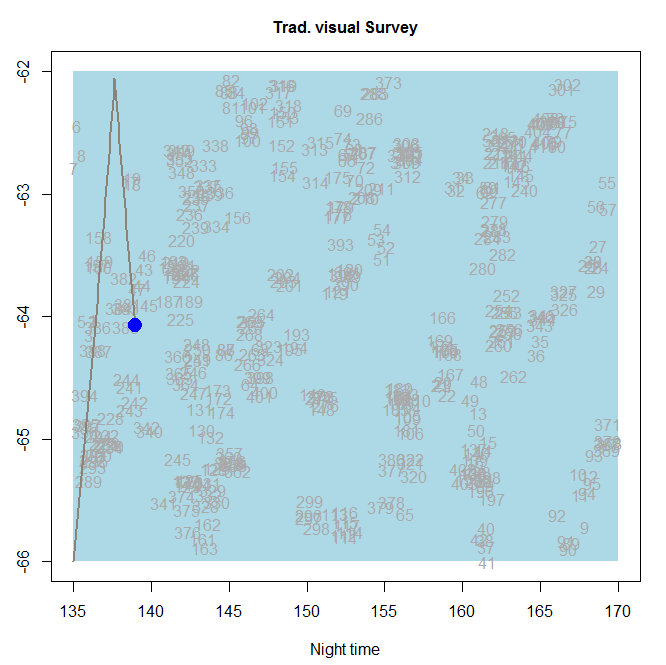 |

1. If you are running on a multicore Linux machine, there are parallel versions *fn.simulationP* and *fn.simulation.sensitivityP*. [↑](#footnote-ref-1)
