# Supplementary material for: A Simulation Study of Acoustic-Assisted Tracking of Whales for Mark-Recapture Surveys
Source: PLoS One. 2014 May 14;9(5):e95602. doi: 10.1371/journal.pone.0095602 (PMC4020746; doi:10.1371/journal.pone.0095602)
Supplement: Appendix S2 — Further detail on movement models. (DOCX) [file pone.0095602.s003.docx]

## **Appendix S2**: Further detail on movement models

Animal movement, especially for whales, can be very complex, depending on physical, biological and social interactions. As little is known, simple models are most appropriate. Excluding the option of assuming no movement, there are three whale movement models currently available in the simulation:

### 1. Simple movement

In this setting, each pod moves independently based on a correlated random walk [1]), as per Fig. 1 in the main paper. This would ultimately result in a uniform distribution of groups. The equations for movement in terms of step size (*S*) and direction bearing (*β*) for whale *i* at time *t*+1 are

$$S_{i,t+1}=gamma(\mu_{S}, \sigma_{S})$$

$$\beta_{i,t+1}=\beta_{i,t+1,}+VonMises( 0, 1/\sigma_{\beta})$$

where gamma(*a*,*b*) is the gamma distribution with mean, *a*, and standard deviation, *b*; $\mu_{S}$, $\sigma_{S}$ are the mean and standard deviation of the step sizes, and $\sigma_{\beta}$ is analogous to the standard deviation of the bearing. In the Antarctic blue whale example in the paper, these are given in Table S2.1. These values were simply for exploration purposes and many were derived from simple experimentation, choosing values that resulted in plausible group movement.

#### **Table S2.1:** Parameters for simple independent movement model.

| **Parameter** | **Antarctic Blue whale Values** | **NZ Sperm whale Values** |
| --- | --- | --- |
| Step size ($\mu_{S}$, $\sigma_{S}$) | 4.5, 0.2 | 5.04, 0.2 |
| Direction ($\sigma_{\beta}$) | 0.02 | 0.02 |

### 2. Clumped movement

This is a simple approach to producing non-uniform distribution of groups. In this setting, clumps of groups are assigned to super clumps that move in a similar fashion. The equations for movement in terms of step size $S_{j}^{(c)}$and direction bearing$\beta_{j}^{\left( c \right)}$ for super clump *j*, at time *t*+1 are a simple correlated random walk

$$S_{j,t+1}^{(c)}={gamma(\mu_{s}^{(c)}}_{,}\sigma_{s}^{(c)})$$

$$\beta_{j,t+1}^{\left( c \right)}=\beta_{j,t}^{\left( c \right)}+VonMises( 0, 1/\sigma_{\beta}^{(c)})$$

where $\mu_{s}^{(c)}$, $\sigma_{s}^{(c)}$ are the mean and standard deviation of the super clump step length, and $\sigma_{\beta}^{(c)}$ is analogous to the standard deviation of the bearing of clump movement. Then an individual group *i* in clump *j* moves according to

$$S_{i,t+1}={gamma(S_{j,t+1}^{(c)}}_{,}\sigma_{S})$$

$$\beta_{i,t+1}=\beta_{j,t+1}^{\left( c \right)}+VonMises( 0, 1/\sigma_{\beta})$$

The values used in the paper are given in Table S2.2.

#### **Table S2.2:** Parameters for simple clumped movement model.

| **Parameter** | **Antarctic Blue whale Values** | **NZ Sperm whale Values** |
| --- | --- | --- |
| **Clump movement** |  |  |
| Clump step size ${(\mu_{s}^{(c)}}_{,}\sigma_{s}^{(c)})$ | 4.5, 1 | 5.0 1.0 |
| Direction ($\sigma_{\beta}^{(c)}$) | 0.05 | 0.05 |
| **Group movement** |  |  |
| Step size $(\sigma_{s})$ | 0.3 | 0.3 |
| Direction ($\sigma_{\beta}$) | 0.1 | 0.1 |

### 3. Herd movement

This approach uses a state space model, which is based on the simulation framework presented in [2]. There are two possible states for each whale group: herd movement, where the group heads toward the herd centroid, and solitary movement, where the group heads off independently (i.e. searching foraging). The groups switch between these states in a Markovian process based on a specified transition matrix (e.g. Table S2.3).

#### **Table S2.3:** Parameters for herd movement model.

| **Parameter** | **Antarctic Blue whale Values** |
| --- | --- |
| **Herd Movement State** |  |
| Step size (${(\mu_{s}^{(c)}}_{,}\sigma_{s}^{(c)}$) | 30, 15 |
| Direction ($\sigma_{\beta}^{(c)}$) | 2.5 |
| **Solitary Group Movement State** |  |
| Step size (gamma mean, sd) | 30, 15 |
| Direction (Von Mises concentration) | 2.5 |
| **Transition Matrix** |  |
| Step size (gamma mean, sd) | $\left( \begin{matrix} 0.95 & 0.05 \\ 0.16 & 0.85 \end{matrix} \right)$ |

References

1. Turchin P (1998) Quantitative analysis of movement: measuring and modeling population redistribution in animals and plants: Sinauer Associates, Sunderland, MA, USA.

2. Langrock R, Hopcraft JGC, Blackwell PG, Goodall V, King R, et al. (2014) Modelling group dynamic animal movement. Methods in Ecology and Evolution.
