# Supplementary material for: A Simulation Study of Acoustic-Assisted Tracking of Whales for Mark-Recapture Surveys
Source: PLoS One. 2014 May 14;9(5):e95602. doi: 10.1371/journal.pone.0095602 (PMC4020746; doi:10.1371/journal.pone.0095602)
Supplement: Appendix S3 — Further information on whale vocalisation/dive model. (DOCX) [file pone.0095602.s004.docx]

## **Appendix S3**. Further information on whale vocalisation/dive model

### Dive Pattern

Groups of whales are typically only available to visual teams when the animals are at the surface. The simulation takes as an argument the mean dive length, and its associated standard deviation, and the mean and standard deviation of surface time. The simulation then generates a sequence of random lengths of diving for each group.

In the case of Antarctic blue whales, the time scale is such that any group detected acoustically would be available upon encounter with the survey vessel. Thus, until further data on surface times are available, these whales are assumed to be available at all times.

### Vocalisation

There are three options for simulating group vocalisation:

**Linked** – As per sperm whales, the animals’ vocalisation is directly linked to the dive pattern. The simulation allows the specification of the proportion of the dive that the whale vocalizes, e.g. 0.8 equates to the animal vocalizing during 80% of the dive.

**Independent** – The simulation takes as an argument, the mean song length and standard deviation, and mean and standard deviation of silence time. The simulation then generates a sequence of random lengths of vocalisation for each group.

**Always** – When the time scale of tracking is much larger than the frequency of singing, the ‘group’ can be set to ‘always singing’. (The probability of the group eventually going silent can be included in the overall probability of abandoning tracking).

There is also the ability to specify what proportion of the groups vocalise, for example if only males vocalise. Note: This is the proportion of groups not individuals. Group vocalising rate can be roughly approximated if a number of assumptions are made. Assuming that the makeup of groups is completely random with regard to sex, and that only and all males vocalise continuously, then

$$\Pr\left( Group Vocalising \right)=1-{(1-Pr(Indiv. Vocalising))}^{\mathbb{E(}Grp. Size)}$$

We must emphasize that these assumptions are not likely to be valid in a real-world setting, and this is an approximation in lieu of any information— it is, therefore, advisable to conduct a sensitivity study (as per the main paper) to gain some insight into the uncertainty. As more data is available on vocalising rates and characteristics, group sex ratios and the number of incidental sightings, these estimates could be improved.
