# Supplementary material for: A Simulation Study of Acoustic-Assisted Tracking of Whales for Mark-Recapture Surveys
Source: PLoS One. 2014 May 14;9(5):e95602. doi: 10.1371/journal.pone.0095602 (PMC4020746; doi:10.1371/journal.pone.0095602)
Supplement: Appendix S4 — IDCR/SOWER calculations for Antarctic blue whales. (DOCX) [file pone.0095602.s005.docx]

## **Appendix S4. IDCR/SOWER calculations for Antarctic blue whales**

From the IDCR/SOWER data [1], we can estimate densities of Antarctic blue whale during the period of our ‘future’ survey. There are number of aspects that must be considered:

1. As our simulations are based on whale groups. not individual animals, all densities must be calculated in terms of groups.
2. As long as the population has no underlying spatial structure, and there is an equal probability of sampling any whale, there is no need for a mark-recapture survey to cover all latitudes. Instead, the survey could focus on the higher density areas, putatively around 0-200km from the summer ice edge.
3. IDCR/SOWER data in this region suggests there are around 0.00023 groups of Antarctic blue whales per km^2^. These data correspond to 1998, the mid-point of the third IDCR/SOWER circumpolar survey.
4. Our potential future survey will be conducted 2013-2025. If we assume density of groups scales linearly with population size, we can extrapolate or predict on circumpolar abundance on Antarctic blue whale throughout 2013-2025 using exponential growth based on the range of population growth rates [1].
5. In 2008, the IWC agreed upon a conservative estimate of 6.4% (95% credibility interval of 2.4-8.4) for the growth rate of circumpolar abundance of Antarctic blue whales [2]). To reflect additional uncertainty incurred by forward projection, we consider potential growth rates between 0% and 10%.

This yields approximate estimates of group densities at 2013 to be between 0.0006, and 0.0011 groups per km^2^ (see Fig. S4.1). However, since there is a great deal of uncertainty around all of the parameters, the uncertainty around these estimates is large.


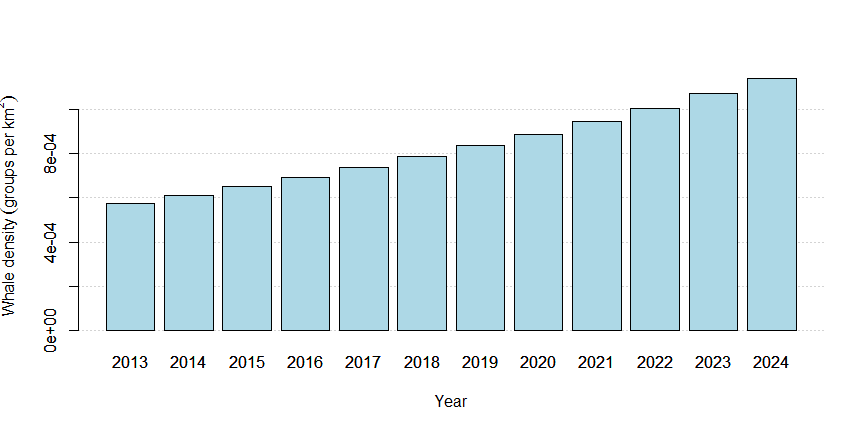


#### **Fig S4.1:** Predicted densities for the purpose of the simulation

IDCR/SOWER data on observed weather conditions was used in the simulation by sampling random windows from the effort database. Considering point 2 above, we sampled weather from effort data collected within 0-200km of the ice edge.

### **References**

1. Branch TA (2007) Abundance of Antarctic blue whales south of 60ºS from three complete circumpolar sets of surveys. Journal of Cetacean Research and Management 9: 253-262.

2. International Whaling Commission. (2009) Report of the Scientific Committee. Annex H. Report of the sub-committee on other Southern Hemisphere whale stocks. *Journal of Cetacean Research and Management. (Suppl.),* **11**, 220-247.
